# A Pilot Evaluation of the Adequacy of Prenatal Vitamins to Cover Dietary Deficits During Pregnancy and Lactation

**DOI:** 10.1002/rfc2.70012

**Published:** 2025-01-02

**Authors:** Chase K. Smith, Emily E. Fay, Sue L. Moreni, Jennie Mao, Mary F. Hebert

**Affiliations:** 1 Departments of Pharmacy, University of Washington, Seattle, Washington, USA; 2 Departments of Obstetrics and Gynecology, University of Washington, Seattle, Washington, USA

**Keywords:** food and nutrient intake, maternal nutrition, prenatal vitamins, supplement evaluation

## Abstract

**Objectives:**

The objectives of this pilot survey were (1) to compare dietary intakes during three survey windows (25–28 weeks gestation, 28–32 weeks gestation and ≥ 3 months postpartum) with the National Institutes of Health, Office of Dietary Supplements' (NIH:ODS) established recommendations for pregnant and lactating women, and (2) to evaluate the general adequacy of commonly used prenatal vitamins (PVs) to cover the identified deficits in dietary intake.

**Methods:**

In this longitudinal pilot survey, 39 healthy consented women, aged 18–50 years with singleton pregnancies and pre-pregnancy BMI < 30.0 kg/m^2^ were included. Items from self-reported dietary intakes during three survey windows were converted to nutritional content using Fooducate (LLC), a public database. Three-day mean intakes per survey window per subject were compared with NIH:ODS recommendations to determine dietary deficits. Eight commonly utilised PVs (identified via web searches of common prenatal vitamins and their availability in local stores) were evaluated for adequacy in correcting each dietary deficit.

**Results:**

Nutrients that were ≥ 30% higher than the recommended RDA/AI were carbohydrates, sodium, vitamin A, and vitamin C in the first survey window; carbohydrates, sodium, and vitamin A in the second survey window; and sodium and iron in the third survey window. Nutrients that were ≥ 30% *lower* than the RDA/AI were potassium, vitamin D, and iron in the first survey window; potassium, vitamin D, and iron during the second survey window; and dietary fibre, potassium, vitamin C, and vitamin D in the third survey window. None of the evaluated PVs corrected all deficits, but two were close to the goal and only one corrected all vitamin D deficits.

**Conclusion:**

Women who are or are planning to become pregnant should be educated on dietary recommendations during pregnancy and lactation, ideally such that supplements become unnecessary. However, it remains good practice to carefully consider prenatal vitamin content before selection.

## 1 ∣ Introduction

To maintain adequate maternal, foetal, and neonatal health during pregnancy and lactation, appropriate nutrition is necessary [1-4]. Prenatal vitamins (PVs) are widely used to provide balance of a mother's diet to maintain overall health. Today, numerous PVs are available through prescription and over the counter, leaving mothers with the arduous task of finding the PV that provides them with the best benefit. After several days of extensive PubMed and Google Scholar literature searches using the search terms of “evaluation” with “prenatal vitamins” and “adequacy” with “prenatal vitamins,” few studies have evaluated PVs for their ability to offset identified dietary deficiencies. Wierzejska sought to evaluate PVs compared to recommended amount used for supplementation made by the 2014 Polish Gynecological Society and 2020 Polish Society of Gynecologists and Obstetricians [5]. Adams et al. sought to provide an evidence base approach to determine “optimal” PV [6]. However, these studies did not evaluate the adequacy of PVs in relation to intakes measured in a population. There is limited data available in the evaluation of the adequacy of PVs in relation to population dietary deficits and established recommended daily allowances (RDAs) and adequate intakes (AIs) such as those published by The National Institutes of Health, Office of Dietary Supplements (NIH:ODS) for women during pregnancy and lactation [7]. These recommendations serve as guidelines for the maintenance of maternal, foetal, and neonatal health (Table 1).

The objectives of this pilot survey were to: (1) compare dietary intakes during 25–28 weeks gestation, 28–32 weeks gestation and ≥ 3 months postpartum with the National Institutes of Health, Office of Dietary Supplements' (NIH:ODS) established adequate intakes (AIs) and recommended dietary allowances (RDAs) for pregnant and lactating women to identify dietary deficits; and (2) to determine the general adequacy of commonly used prenatal vitamins' (PVs) ability to reduce identified deficits in dietary intake.

## 2 ∣ Materials and Methods

### 2.1 ∣ Materials

Materials include the use of the publicly available nutrition database from Fooducate (Fooducate Ltd. San Francisco, CA).

### 2.2 ∣ Survey Participants

The data utilised in this pilot survey originated from a separate study evaluating regulators of CYP2D6 activity in extensive metabolizers during pregnancy and postpartum [8]. Women between 12 and 27 weeks gestation were recruited from a large urban medical centre, University of Washington Medical Centre, and its satellite clinics, as well as via recruitment flyers posted around the Seattle area. Gestational ages of the subjects were determined based on last menstrual period (LMP) using a pregnancy wheel. In the accompanying survey, healthy women, with singleton pregnancies and 18–50 years of age were enroled, of which available dietary intake data was extracted. Women who were poor or extensive metabolizers of CYP2D6 were excluded from the parent study. Exclusion criteria included current obesity (prepregnancy BMI > 30.0 kg/m^2^), diabetes, kidney disease, liver disease, asthma, chronic obstructive pulmonary disease, smoking, fever or cough, treatment for mental illness as well as receiving medications or dietary supplements known to interact with cytochrome CYP2D6 or CYP3A. All healthy pregnant women in this study received clinical care counselling on appropriate dietary intake, foods to avoid, appropriate weight gain and prenatal vitamins during pregnancy and lactation as well as being provided with an eight-page information sheet with more detailed information on these topics. Subjects were also asked to report if they were currently taking prenatal vitamins during the first study day. The data utilised in this pilot survey was obtained from another study that underwent ethics review and received approval from the University of Washington Institutional Review Board. All subjects provided written informed consent before participation.

### 2.3 ∣ Dietary Food Log Collection

Nutritional content of dietary intake was determined during three survey windows: 25–28 weeks gestation, 28–32 weeks gestation, and ≥ 3 months postpartum. Pregnancy survey windows were separated by 3–4 weeks.

Subjects participating in the pilot survey submitted self-recorded dietary logs of all food and drink with their best estimates of their intake amounts each day. Subjects were asked to record all food and beverage intake along with the amount/volume of each for three consecutive days in each survey window in a food log. Basic instructions on how to determine and record the amount were given to the subjects. The food log was a simple list of logging of all food and drink with manufacturer under each section including the amount or volume of each item. Food logs consisted of labelled sections of mealtimes denoted as breakfast, morning snack, lunch, after lunch snack, dinner, and after dinner snack to aid the subject in collecting dietary intake by breaking down the day into chunks. Each item in the dietary food log was converted by the researchers to the corresponding amount of each respective nutrient (nutritional content) using Fooducate, a publicly available nutrition database (Fooducate LTD. San Francisco, CA, USA) [9]. Individual daily dietary consumption was determined by averaging dietary intake over three consecutive days during each survey window.

### 2.4 ∣ Data Analysis

Eleven nutrients with NIH:ODS published DRI values (calories, carbohydrates, protein, dietary fibre, sodium, potassium, vitamin A, vitamin C, calcium, vitamin D, and iron) were compared with 3-day mean intake during each survey window for each subject. Mean, standard deviation, range, and a 95% confidence interval were calculated for each nutrient in each survey window.

A ≥ 30% difference from the RDA/AI was used to identify those with potentially clinically significant differences from nutritional guidelines. The focus of this analysis is on what we believe to be a clinically relevant break-point, which was set a priori at 30% difference from RDA/AI. To determine the break point, we started with what is clinically relevant for pharmaceutical compounds. In the case of medications, FDA guidance uses 80%–125% as the no effect boundary [10]. For this study, given that the evaluation is for nutrients, we relaxed this to a ≥ 30% difference from RDA/AI as our clinically relevant break-point.

For nutrient intakes below RDA/AI, the difference between the mean 3-day intake and RDA/AI (i.e., deficits) in each survey window were compared to eight commonly used, commercially available, prenatal vitamins (PVs), designated as PV-A through PV-H (Table 2), to assess for appropriateness of content based on nutritional needs of each healthy pregnant and lactating woman to achieve recommended dietary intake. An arbitrary mark of ≥ 80% of the recommended intake was utilised to signify the percentage of subjects whose dietary intake was close to the RDA/AI. PVs were identified by those available in local stores (Seattle, WA) and through web searches of ‘popular prenatal vitamins’. Five nutrients (vitamins A, C, and D; calcium; and iron) were evaluated because they appear in both the data collected from Fooducate and on PV labels. The ability of each PV to reduce a deficit was determined by adding its content to each subject's 3-day mean intake deficit and comparing it with RDA/AI from NIH:ODS during each survey window.

## 3 ∣ Results

### 3.1 ∣ Demographics

Eighty-one female subjects were enroled in the CYP2D6 study, of which 47 completed all three study windows and complete dietary intake data was available for 39 subjects (aged 25–41 years). Race and ethnicity were self-reported. Thirty subjects (76.9%) identified as White, four subjects (10.3%) Black, four subjects (10.3%) Asian, and one subject (2.6%) Pacific Islander. None of the subjects reported being Hispanic or Latina. The mean (SD) age was 32 (3.6) years with a range of 25–41 years., 11 subjects were 20–29 years old, 27 subjects 30–39 years old, and 1 subject ≥ 40 years old (Table 3). Height groups are also shown in Table 3. The subject pool included no vegans, four vegetarians, and 35 omnivorous dietary habits. Bodyweight changes were also measured, the mean (SD) weight gain from pre-pregnancy to ≥ 3 months postpartum was 1.7 (4.3) kg (95% CI: 0.45, 3.04). During the first survey day (25–28 weeks gestation), subjects were interviewed and they all reported they were taking one prenatal multivitamin once a day. The brands of prenatal vitamins were not collected.

Several pre-existing conditions were identified including, heartburn, three cases of hypothyroidism, three cases of anaemia, long QT syndrome, endometriosis, and an ebstein anomaly. Of the primiparous and multiparous women, notable maternal/foetal/neonatal complications from past pregnancies include preterm labour, hyperemesis gravidarum, pre-eclampsia, foetal growth restriction, foetal heart block, placenta previa, and accessory lobe of placenta with velamentous cord insertion. The duration of pregnancy was also recorded as whole weeks (a duration of 38 weeks and 3 days is recorded as 38 weeks, e.g.), two subjects completed ≤ 35 weeks, one subject only completed 36 weeks, five subjects completed 37 weeks, seven subjects completed 38 weeks, 11 subjects completed 39 weeks, 13 subjects completed ≥ 40 weeks and the mean (SD) pregnancy duration was 38 (4.5) whole weeks (95% CI: 36.70, 39.61) (Table 3).

In this pilot study, identified pregnancy/foetal/neonatal complications occurring in the subject population included heartburn, gestational hypertension, congenital hypothyroidism, foetal growth restriction, maternal hyperemesis, gestational diabetes, and pre-eclampsia. In the US State of Washington, where this study took place, all women who become pregnant are insured by the state for the duration of the pregnancy and through 6-weeks post-partum.

### 3.2 ∣ Dietary Intake Versus Recommended Intake

**FIGURE 1 ∣:**
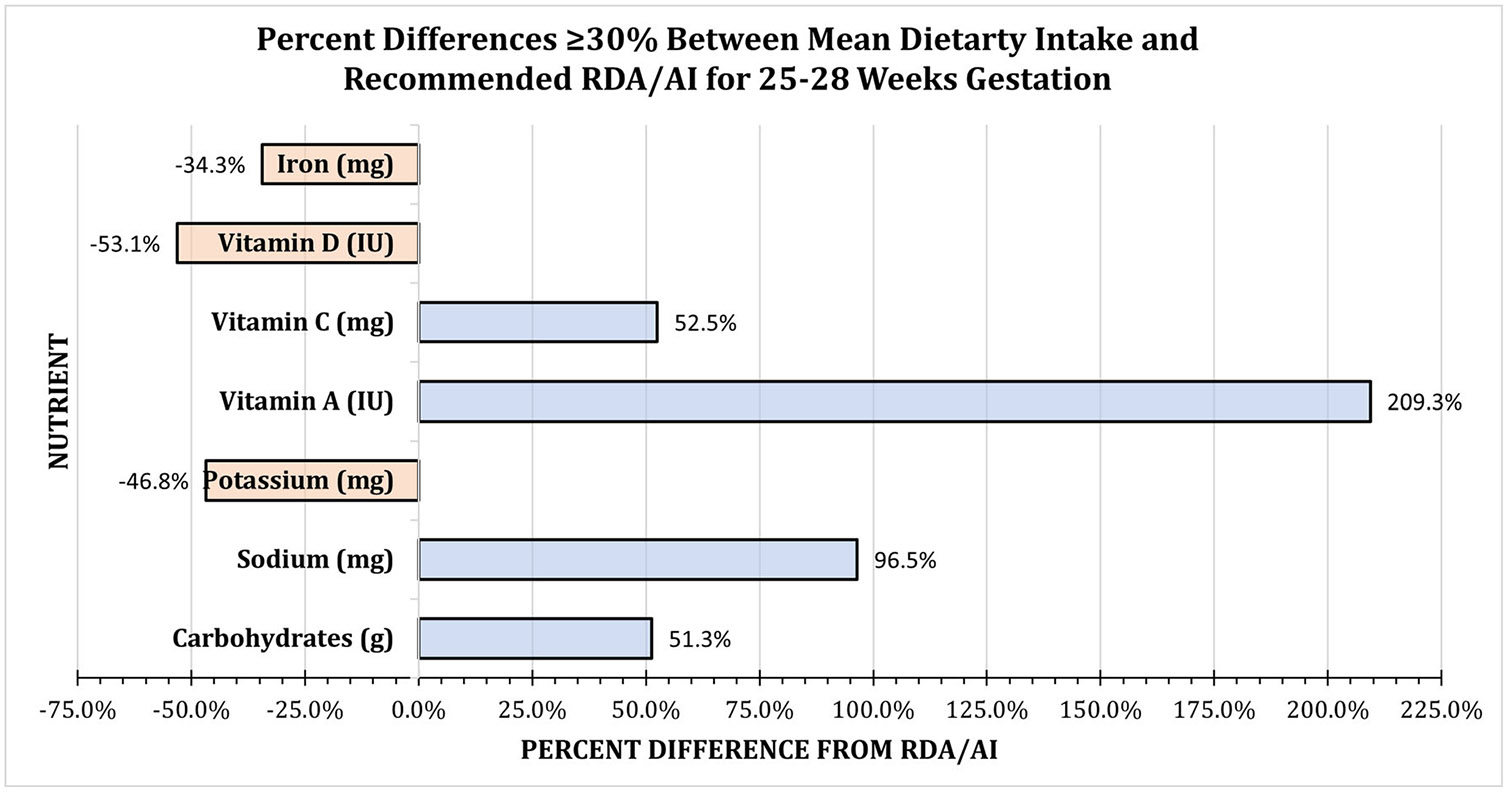
Mean dietary intake with percent differences ≥ 30% from the recommended RDA/AI for 25–28 weeks Gestation. Nutrients with a percent difference ≥ 30% higher than the RDA/AI were vitamin C (52.5%), vitamin A (209.3%), sodium (96.5%), and carbohydrates (51.3%); and ≥ 30% lower were iron (34.3%), vitamin D (53.1%), and potassium (46.8%) (Table 1A).

During the 25–28 weeks gestation survey window, mean intakes of four nutrients were ≥ 30% higher than the RDA/AI. The calculated mean for carbohydrates of 265 g (95% CI: 243.24, 286.26) was 51.3% higher, for sodium of 2947 mg (95% CI: 2681.53, 3212.53) was 96.5% higher, for vitamin A of 7940 IU (95% CI: 4789.4, 11090.21) was 209.3% higher, and for vitamin C of 130 mg (95% CI: 106.3, 152.97) was 52.5% higher. Mean intakes of three nutrients were ≥ 30% lower than the RDA/AI. The calculated mean for potassium of 1543 mg (95% CI: 1212.11, 1773.54) was 46.8% lower, for vitamin D of 281 IU (95% CI: 161.89, 400.92) was 53.1% lower, and for iron of 18 mg (95% CI: 14.85, 20.60) was 34.3% lower (Figure 1, Table 1A).

**FIGURE 2 ∣:**
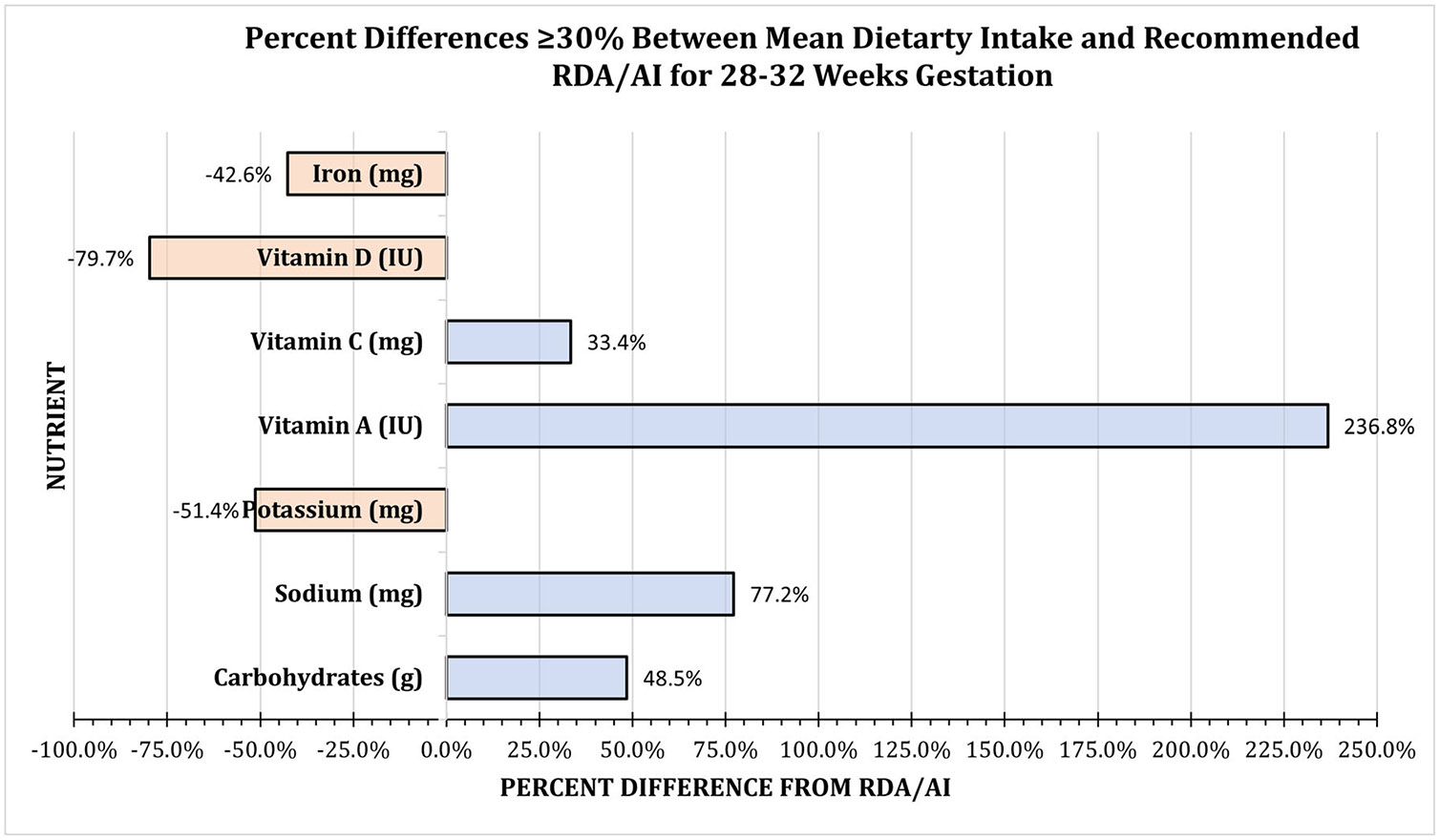
Mean dietary intake with percent differences ≥ 30% from the recommended RDA/AI for 28–32 weeks gestation. Nutrients with a percent difference ≥ 30% *higher* than the RDA/AI were vitamin C (33.4), vitamin A (236.8%), sodium (77.2%), and carbohydrates (48.5%); and ≥ 30% *lower* were iron (42.6%), vitamin D (79.7%), and potassium (51.4%) (Table 1B).

During the 28–32 weeks gestation survey window, mean intakes of four nutrients were ≥ 30% higher than the RDA/AI. The calculated mean for carbohydrates of 260 g (95% CI: 225.14, 294.64) was 48.5% higher, for sodium of 2658 mg (95% CI: 2360.53, 2956.12) was 77.2% higher, for vitamin A of 8644 IU (95% CI: 4483.07, 12804.65) was 236.8% higher, and for vitamin C of 113 mg (95% CI: 86.5, 140.36) was 33.4% higher. Mean intakes of three nutrients were ≥ 30% lower than the RDA/AI. The calculated mean for potassium of 1410 mg (95% CI: 1173.52, 1645.84) was 46.8% lower, for vitamin D of 122 IU (95% CI: 63.29, 180.59) was 79.7% lower, and for iron of 15 mg (95% CI: 13.13, 17.84) was 42.6% lower (Figure 2, Table 1B).

**FIGURE 3 ∣:**
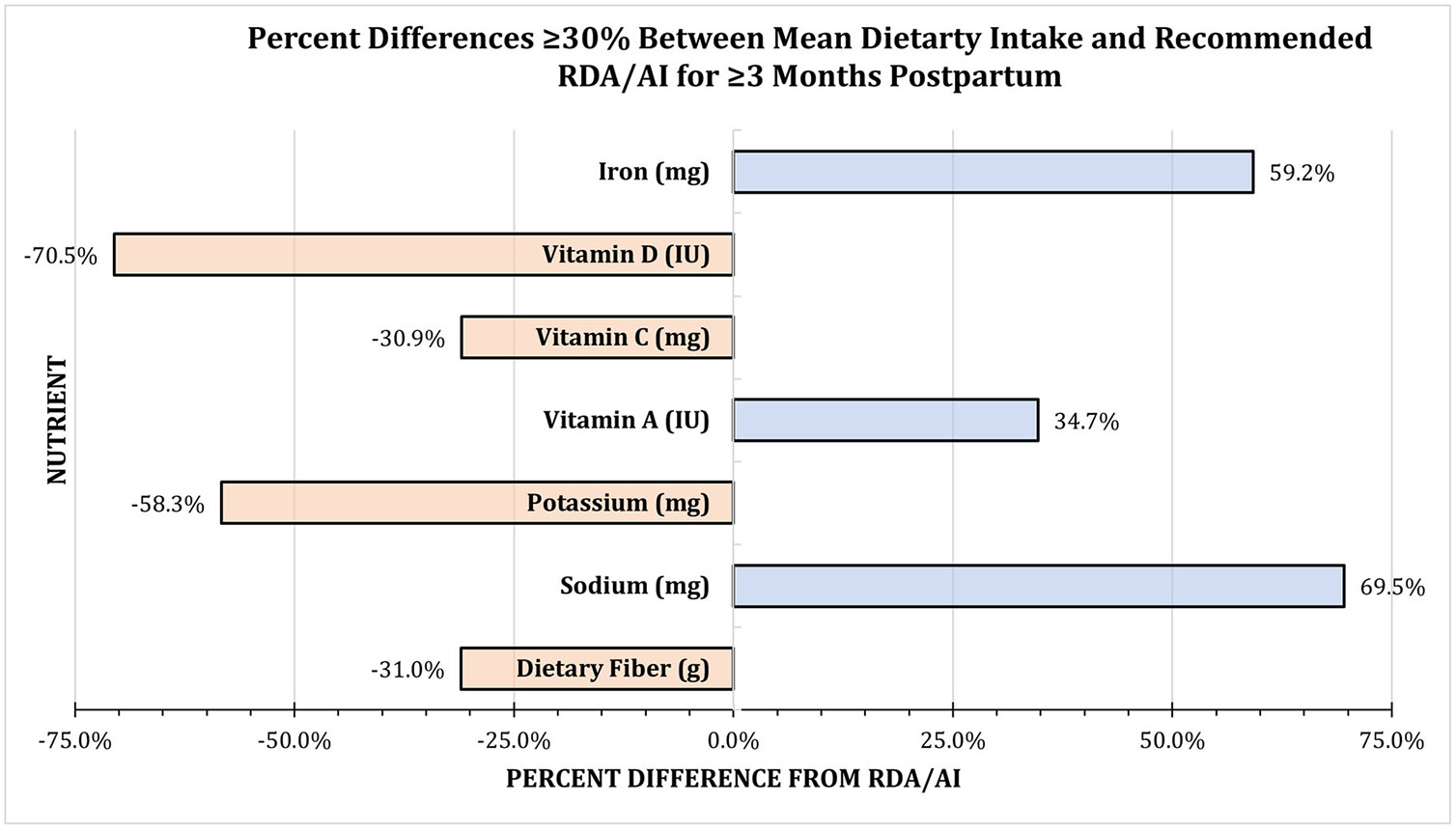
Mean dietary intake with percent differences ≥ 30% from the recommended RDA/AI for ≥ 3 months postpartum. Nutrients with a percent difference ≥ 30% *higher* than the RDA/AI were iron (59.2%), vitamin A (34.7%), and sodium (69.5%); and ≥ 30% *lower* were vitamin D (70.5%), vitamin C (30.9%), potassium (58.3%), and dietary fibre (31.0%) (Table 1C).

During the ≥ 3 months postpartum survey window, mean intakes of three nutrients were ≥ 30% higher than the RDA/AI. The calculated mean for sodium of 2543 mg (95% CI: 2241.44, 2844.45) was 69.5% higher, for vitamin A of 5836 UI (95% CI: 4527.05, 7144.46) was 34.7% higher, and for iron of 15 mg (95% CI: 11.52, 17.14) was 59.2% higher. Mean intakes of four nutrients were ≥ 30% lower than the RDA/AI. The calculated mean for dietary fibre of 20 g (95% CI: 17.26, 22.75) was 31% lower, for potassium of 1167 mg (95% CI: 987.69, 1345.56) was 58.3% lower, for vitamin C of 83 mg (95% CI: 59.88, 105.85) was 30.9% lower for vitamin D of 177 IU (95% CI: 77.71, 275.77) was 70.5% lower (Figure 3, Table 1C).

### 3.3 ∣ Prenatal Vitamin Adequacy

#### 3.3.1 ∣ Vitamin A

At 25–28 weeks gestation, 17.9% of subjects were below vitamin A recommendations for intake, but of those with deficits, 71.4% consumed ≥ 80% of vitamin A RDA. PV-A, PV-B, PV-C, PV-D, PV-F, and PV-H covered 100% of the deficits in all subjects. PV-E left 28.6% of subjects with some portion of their deficits uncorrected. PV-G had no vitamin A (Table 4A).

At 28–32 weeks gestation, 23.1% of subjects were below vitamin A recommendations for intake, but of those with deficits, 44.4% consumed ≥ 80% of vitamin A RDA. PV-A, PV-B, PV-C, PV-D, PV-F, and PV-H covered 100% of the deficits in all subjects. PV-E left 11.1% of subjects with some portion of their deficits uncorrected. PV-G had no vitamin A (Table 4B).

At ≥ 3 months postpartum, 46.2% of subjects were below vitamin A recommendations for intake, but of those with deficits, 16.7% consumed ≥ 80% of the vitamin A RDA. PV-B was the only vitamin to cover 100% of the deficits in all subjects. PV-A left 22.2% of subjects with vitamin A deficits uncorrected, PV-C left 11.1%; PV-D, PV-F, and PV-H left 44.4%; and PV-E left 77.8% of subjects who had deficits uncorrected. PV-G had no vitamin A (Table 4C).

#### 3.3.2 ∣ Vitamin C

At 25–28 weeks gestation, 35.9% of subjects were below vitamin C recommendations for intake, but of those deficits, 42.9% consumed ≥ 80% of the vitamin C RDA. PV-A, PV-C, and PV-F covered 100% of the deficits in all subjects. PV-B left 14.3%, PV-D left 7.1%, PV-E left 50.0%, and PV-H left 14.3% of subjects with some portion of their vitamin C deficits uncorrected. PV- G had no vitamin C (Table 4A).

At 28–32 weeks gestation, 46.2% of subjects were below vitamin C recommendations for intake, but of those with deficits, 22.2% consumed ≥ 80% of the vitamin C RDA. PV-A, PV-C, and PV-F covered 100% of deficits in all subjects. PV-B left 33.3%, PV-D left 11.1%, PV-E left 77.8%, and PV-H left 27.8% of subjects with some portion of their vitamin C deficits uncorrected. PV-G had no vitamin C (Table 4B).

At ≥ 3 months postpartum, 79.5% of subjects were below vitamin C recommendations for intake, but of those subjects with deficits, 9.7% consumed ≥ 80% of the vitamin C RDA. PV-F was the only PV to covered 100% of the vitamin C deficits in all subjects. PV-A left 29.0%, PV-B left 64.5%, PV-C left 12.9%, PV-D left 51.6%, PV-E left 93.5%, and PV-H left 58.1% of subjects with some portion of their deficits uncorrected. PV- G had no vitamin C (Table 4C).

#### 3.3.3 ∣ Calcium

At 25–28 weeks gestation, 61.5% of subjects were below calcium recommendations for intake, but of those with deficits, 29.2% consumed ≥ 80% of the calcium RDA. None of the evaluated PVs covered 100% of the deficits in all subjects. PV-A and PV-C left 79.2%, PV-B left 70.8%, PV-D and PV-H left 91.7%, and PV-F and PV-G left 100% of subjects with some portion of their deficits uncorrected. PV-E had no calcium (Table 4A).

At 28–32 weeks gestation, 61.5% of subjects were below calcium recommendations for intake, but of those with deficits, 25.0% consumed ≥ 80% of the calcium RDA. None of the evaluated PVs covered 100% of the deficits in all subjects. PV-A and PV-B left 75.0%, PV-C left 79.2%, PV-D left 87.5%; and PV-F, PV-G, and PV-H left 100% of subjects with some portion of their deficits uncorrected. PV-E had no calcium (Table 4B).

At ≥ 3 months postpartum, 74.4% of subjects were below calcium recommendations for intake, but of those with deficits, 31.0% consumed ≥ 80% of the calcium RDA. None of the evaluated PVs covered 100% of the deficits in all subjects. PV-A left 79.2%, PV-B left 69.0%, PV-C left 79.3%, PV-D left 86.2%; and PV-F, PV-G, and PV-H left 100% of the subjects with some portion of their deficits uncorrected. PV-E had no calcium (Table 4C).

#### 3.3.4 ∣ Vitamin D

At 25–28 weeks gestation, 84.6% of subjects were below vitamin D recommendations for intake, but of those with deficits, 3.0% consumed ≥ 80% of the vitamin D RDA. PV-A, PV-C, PV-D, PV-E, PV-F, and PV-G covered 100% of the deficits in all subjects. PV-B left 72.7% and PV-H left 3.0% of the subjects with some portion of their deficits uncorrected (Table 4A).

At 28–32 weeks gestation, 97.4% of subjects were below vitamin D recommendations for intake, and of those with deficits 0% consumed ≥ 80% of the vitamin D RDA. PV-A, PV-C, PV-D, PV-E, PV-F, and PV-G covered 100% of the deficits in all subjects. PV-B left 86.8% and PV-H left 2.7% of the subjects with some portion of their deficit uncorrected (Table 4B).

At ≥ 3 months postpartum, 94.9% of the subjects were below vitamin D recommendations for intake, and of those with deficits, 0% consumed ≥ 80% of the vitamin D RDA. PV-A, PV-C, PV-D, PV-E, PV-F, and PV-G covered 100% of the deficits in all subjects. PV-B left 78.4% and PV-H left 2.7% of the subjects with some portion of their deficits uncorrected (Table 4C).

#### 3.3.5 ∣ Iron

At 25–28 weeks gestation, 87.2% of subjects were below iron recommendations for intake, but of those with deficits, 11.8% consumed ≥ 80% of the iron RDA. PV-A, PV-B, PV-C, PV-D, and PV-H covered 100% of the deficits in all subjects. PV-E, PV-F, and PV-G had no iron (Table 4A).

At 28–32 weeks gestation, 92.3% of subjects were below iron recommendations for intake, but of those with deficits, 5.6% consumed ≥ 80% of the iron RDA. PV-A, PV-B, PV-C, PV-D, and PV-H covered 100% of the deficits in all subjects. PV-E, PV-F, and PV-G had no iron (Table 4B).

At ≥ 3 months postpartum, 28.2% of subjects were below iron recommendations for intake, but of those with deficits, 63.6% consumed ≥ 80% of the iron RDA. PV-A, PV-B, PV-C, PV-D, and PV-H covered 100% of the deficits in all subjects. PV-E, PV-F, and PV-G had no iron (Table 4C).

## 4 ∣ Discussion

Pregnant and lactating women commonly take PVs, which theoretically promote adequate nutrition as well as maternal, foetal, and neonatal health. The content of PVs is variable. No PVs completely corrected all dietary deficits in the subject population, although PV-A and PV-C appeared to approach this goal. On the other hand, PV-G corrected all vitamin D deficits but did not fully offset any other dietary insufficiencies. While supplementation of micronutrients has been associated with improved maternal-foetal outcomes in undernourished pregnant women and make theoretical sense, the benefits of micronutrient supplementation, other than folic acid, iron, and iodine, in developed countries are still unclear [11].

Vitamin A supplementation during pregnancy requires careful consideration. Most dietary sources of vitamin A are in the carotenoid form (also called provitamin A) and metabolised into active vitamin A (retinol, retinal and retinoic acid) [12]. Both excessive and insufficient amounts of vitamin A during pregnancy can result in complications [13, 14]. Excessive vitamin A intake is associated with increased risk of malformations, particularly during the first 60 days postconception. The extent and type of malformations depend on the concentration of vitamin A and its derivatives and the gestational age at the time of exposure [15-18]. Excessive vitamin A during organogenesis can result in miscarriage as well as anomalies of the urinary tract, central nervous system, and heart [19-22]. Vitamin A intake during pregnancy exceeding recommended amounts is not uncommon in developed countries as was the case for many of the women participating in this pilot survey. This pilot survey took place after completion of organogenesis. We found a number of outliers with > 10 000 IU of vitamin A intake from vegetable sources high in vitamin A (e.g. carrots). This did not produce high concentrations of vitamin A in these subjects, likely due to poor absorption of vitamin A from vegetable sources [23]. In contrast, it is common in low-income countries for pregnant women to have inadequate intake of vitamin A resulting in vitamin A deficiency. Vitamin A deficiency is the leading cause of preventable blindness worldwide [24]. Vitamin A insufficiency can result in blindness as well as affect bone development, the immune system, teeth, hair, functional capacity of reproductive organs and overall normal development of the embryo [24]. Although based on the dietary intake of vitamin A in our survey, vitamin A supplementation would not be necessary during pregnancy, as most subjects achieved the RDA through their diet alone. This recommendation falls counter to our previous report of retinoid concentrations during 28–32 weeks gestation in this same population in which 5 women had vitamin A deficiency (plasma retinol concentration < 0.7 μM) and 48% of the women were vitamin A insufficient (clinically defined as plasma retinol concentration > 0.70 μM but < 1.05 μM) [25]. Further, we found a poor correlation between vitamin A intake and retinoid concentrations during pregnancy. Rothman et al. concluded that 1 in 57 infants born to women who took more than 10 000 IU/day of vitamin A had a malformation attributable to supplementation; however, this study's findings relate solely to vitamin A (retinol), not to beta-carotene, a precursor to vitamin A [16]. The World Health organisation guidance does not recommend vitamin A supplementation for routine antenatal care for the prevention of maternal and infant morbidity and mortality, but does recommend vitamin A supplementation in areas with severe public health issues related to vitamin A deficiency [26]. The area where this pilot survey was conducted (Seattle, WA) does not fall into this category. Interestingly, during the ≥ 3 months postpartum survey window, although almost half of the subjects' intakes were below the RDA, 90% of the women had vitamin A concentrations back to what is considered clinically sufficient [25]. Dietary guidelines for vitamin A during pregnancy and postpartum require further evaluation.

Vitamin D is consumed in the diet as well as being synthesised in sun-exposed skin. Further processing of vitamin D in the liver and kidneys yields 1,25-dihydroxyvitamin D, which promotes the absorption of calcium in the small intestine and aids in bone mineralisation. Even when calcium intake is adequate, insufficient vitamin D will lead to poor calcium absorption [27-29]. Vitamin D deficiency during pregnancy is common and can be associated with reduction in maternal bone mineral density, congenital rickets and fractures in the newborn [30-33]. However, based on infrequent case reports, osteoporosis does not appear to be common. Of note, even in a population with wide-spread vitamin D deficiency, supplementation with vitamin D starting mid-pregnancy to 6 months postpartum did not improve foetal or infant growth [34]. Optimum vitamin D concentrations during pregnancy are unclear and an active area of research. During pregnancy, calcium is transferred from the maternal skeleton to the foetus. Calcium supplementation for pregnant women that consume insufficient calcium in their diets has been associated with modest benefits in preventing pre-eclampsia, preterm birth and improved maternal and infant bone health [33, 35]. In addition, calcium carbonate 1200 mg/day along with iron sulphate 30 mg/day starting in the first trimester to 1 month postpartum in the setting of adequate calcium intake has been associated with a reduction in maternal bone reabsorption [36]. The insufficient dietary calcium and vitamin D during pregnancy described in this pilot survey would be expected to worsen the effects of pregnancy and lactation on maternal bone mineral density, particularly in the low-level sunlight region where the pilot survey was conducted. A Cochrane review examined placebo vs vitamin D alone, with calcium, and with both calcium and other vitamins during pregnancy. They concluded that vitamin D alone during pregnancy likely reduced the risk of pre-eclampsia, gestational diabetes, and low birthweight infants compared to placebo [37].

Iron requirements are higher during pregnancy to compensate for the increased red cell mass, growing foetus and placenta as well as blood loss during delivery [38]. Iron-deficiency anaemia has a prevalence of ~5.7% in pregnant women in the United States [39]. There is a U-shaped relationship between iron concentrations and pregnancy outcomes (i.e., both low and high iron concentrations have been associated with increased risk of complications). Low iron concentrations have been associated with preterm birth, low birth weight, perinatal mortality, low resistance to infections and poor cognitive development [40, 41]. In this pilot survey, almost 90% of subjects did not consume the recommended dietary intake for iron, putting these women at risk for anaemia and its associated complications. In addition, by increasing GI pH, heartburn treatments commonly utilised during pregnancy decrease iron absorption further exacerbating low iron intake. A Cochrane review concluded that iron supplementation reduces the risk of maternal anaemia and iron deficiency in pregnancy [42]. Several studies examined iron deficiency and supplementation during pregnancy [43]. However, we were unable to find studies that quantified dietary iron deficits and PV adequacy. Seligman et al. reported that the amount iron absorbed from many prenatal vitamins that also contain calcium (in the form of calcium carbonate) is lower than with prenatal vitamins without calcium as well as iron supplementation alone. In addition, Dawson et al. reported on the variability in iron absorption across prenatal multivitamin-mineral products [44, 45]. Bradley et al. determined that constipation prevalence among pregnant women is 24%, 26% and 16% during the 1st, 2nd and 3rd trimesters of pregnancy, respectively and 24% at 3 months postpartum [46]. Iron supplementation and inadequate intake of dietary fibre during pregnancy and lactation may exacerbate the already increased prevalence of constipation in these women [47]. Furthermore, C. Zhang et al. and X. Zhang et al. concluded that a diet low in fibre intake and high in glycemic load is associated with an increased risk of developing gestational diabetes mellitus [48, 49].

Limitations of this pilot survey include small subject sample size, self-reporting of dietary intake with dietary food logs vitamin intake potentially influencing dietary consumption, Fooducate LLC's ongoing updating of their nutrient database, and limited number of nutrients evaluated. It is possible that subjects under- or over-estimated portion size, forgot foods they consumed, or incorrectly identify the brands or types of food consumed during each 3-day survey window. In some cases, foods consumed at eateries did not provide the nutritional content of their food in the detail that this research requires. Subjects may also have knowingly altered their diet knowing it was being evaluated as part of this pilot survey. It is also possible that women considered their PV intake in their food consumption. Fooducate LLC, has continuously updated their database with new food data and may refresh current logged foods with more accurate nutrition facts or update previously missing nutrition facts, which over a long survey will affect the consistency of collected data. Additionally, PVs are used to supplement the intake of important micronutrients such as folate, which was not evaluated in this pilot survey. This survey identifies an important area that requires further research utilising larger datasets and an improved method for recording dietary intakes.

## 5 ∣ Conclusions

In summary, adequate nutrition to maintain maternal, foetal and neonatal health during pregnancy and lactation is important [1-4]. To ensure adequate supplementation with respect to current guidelines, maintaining a balanced diet and evaluating the contents of PVs remains important before product selection.

This pilot survey highlights dietary excess during 25–28 weeks gestation (carbohydrates, sodium, vitamin A, and vitamin C), 28–32 weeks gestation (carbohydrates, sodium, and vitamin A), and ≥ 3 months postpartum (sodium and iron) as well as dietary deficits during 25–28 weeks gestation (potassium, vitamin D, and iron), 28–32 weeks gestation (potassium, vitamin D, and iron), and ≥ 3 months postpartum (potassium, vitamin C, and vitamin D). Furthermore, this pilot survey identifies suboptimal contents of popular PVs when addressing dietary deficits in pregnancy and lactation. These preliminary results signal the need for a larger, more thorough survey to clearly identify patterns of deficits and excesses in the diets of pregnant and lactating women and determine the inadequacies of common prenatal vitamins.

## Figures and Tables

**TABLE 1 ∣:** Published recommended intake (RDA/AI) and reported dietary intake during 25–28 weeks gestation (A), 28–32 weeks gestation (B), and ≥ 3 months postpartum (C). Reported mean dietary intake for each survey window and the respective NIH:ODS recommended intake during each of the survey windows [7].

	(A) 25–28 weeks gestation, *n* = 39				(B) 28–32 weeks gestation, *n* = 39				(C) ≥ 3 Months postpartum, *n* = 39			
Nutrient	RDA/AI	Mean intake (SD)	95% confidence interval	Range	RDA/AI	Mean intake (SD)	95% confidence interval	Range	RDA/AI	Mean intake (SD)	95% confidence interval	Range
**Calories (kcal)**	2140	2057 (506)	(1897.76, 2215.33)	(946–3145)	2252	1966 (532)	(1799.47, 2133.26)	(557–3294)	2130	1828 (624)	(1632.25, 2023.92)	(688–4015)
**Carbohydrates (g)**	175	265 (68)	(243.24, 286.23)	(132–404)	175	260 (111)	(225.14, 294.64)	(27–779)	210	212 (89)	(184.16, 239.73)	(49–505)
**Protein (g)**	71	81 (27)	(72.79, 89.88)	(33–165)	71	72 (23)	(64.49, 79.18)	(23–139)	71	74 (23)	(66.61, 81.18)	(30–136)
**Dietary Fibre (g)**	28	27 (16)	(22.28, 32.43)	(6–87)	28	25 (10)	(22, 28.24)	(6–55)	29	20 (9)	(17.26, 22.75)	(4–43)
**Sodium (mg)**	1500	2947 (846)	(2681.53, 3212.53)	(1319–5461)	1500	2658 (949)	(2360.53, 2956.12)	(1096–5864)	1500	2543 (961)	(2241.44, 2844.45)	(995–5068)
**Potassium (mg)**	2900	1543 (735)	(1312.11, 1773.54)	(547–3945)	2900	1410 (752)	(1173.52, 1645.84)	(65–3288)	2800	1167 (570)	(987.69, 1345.56)	(440–2863)
**Vitamin A (IU)**	2567	7934 (10038)	(4789.4, 11 090.21)	(1080–58 871)	2567	8644 (13257)	(4483.07, 12804.65)	(1256–78 819)	4333	5836 (4170)	(4527.05, 7144.46)	(1052–15 939)
**Vitamin C (mg)**	85	130 (74)	(106.3, 152.97)	(3–316)	85	113 (86)	(86.5, 140.36)	(5–285)	120	83 (73)	(59.88, 105.85)	(1–342)
**Calcium (mg)**	1000	905 (319)	(804.5, 1004.53)	(469–1743)	1000	990 (624)	(794.63, 1186.19)	(83–4034)	1000	801 (382)	(681.51, 921.3)	(91–2040)
**Vitamin D (IU)**	600	281 (381)	(161.89, 400.92)	(0–1544)	600	122 (187)	(63.29, 180.59)	(0–1108)	600	177 (316)	(77.71, 275.77)	(0–1585)
**Iron (mg)**	27	18 (9)	(14.85, 20.6)	(5–41)	27	15 (8)	(13.13, 17.84)	(3–40)	9	14 (9)	(11.52, 17.14)	(2–48)

**TABLE 2 ∣:** Eight evaluated prenatal vitamins.

Evaluated prenatal vitamins	
PV	Prenatal vitamin
A	Nature Made Prenatal + DHA
B	Women's One a Day: Prenatal
C	TheraNatal Complete
D	New Chapter Perfect Prenatal
E	Vitafusion Prenatal Gummies
F	Garden of Life: Mykind Organic Prenatal
G	Ritual: Essential Prenatal
H	RainbowLight: One Prenatal + DHA

**TABLE 3 ∣:** Subject demographics and weight change.

Age (years)	*n*		%	
< 20	0		0.0%	
20–29	11		28.2%	
30–39	27		69.2%	
≥ 40	1		2.6%	
Mean Age (SD) [95% CI]	32 (3.6) [31.10, 33.41]		100%	
Height (m)				
< 1.50	0		0.0%	
1.50–1.59	5		12.8%	
1.60–1.69	19		48.7%	
1.7–1.79	14		35.9%	
1.8–1.89	1		2.6%	
≥ 1.90	0		0.0%	
Mean Height (SD) [95% CI]	1.67 (0.07) [1.65, 1.69]		1%	
	Prepregnancy		≥ 3 months postpartum	
BMI (kg/m^2^)	*n*	%	*n*	%
< 18.5	3	7.7%	1	2.6%
18.5–24.9	27	69.2%	23	59.0%
25–29.9	8	20.5%	15	38.5%
≥ 30	1	2.6%	0	0.0%
Mean Weight (kg (SD) [95% CI])	Prepregnancy	25–28 weeks	28–32 weeks	≥ 3 months Postpartum
	65.7 (10.9) [62.13, 69.20]	71.1 (10.2) [67.83, 74.46]	72.8 (10.5) [69.46, 76.24]	67.4 (9.6) [64.32, 70.51]
Weight Change (Δ kg (SD) [95%CI])	Prepregnancy	25–28 weeks	28–32 weeks	≥ 3 months Postpartum
	5.5 (4.5) [4.01, 6.95]			
		1.7 (1.8) [1.11, 2.30]		
			−5.4 (−4.5) [−6.91, −3.97]	
		1.7 (4.3) [0.36, 3.13]		
Dietary habits	*n*		%	
Vegan	0		0.0%	
Vegetarian	4		10.3%	
Omnivoroius	35		89.7%	
Parity				
Nulliparous	0		0.0%	
Primiparous	22		56.4%	
Multiparous	17		43.6%	
Pregnancy duration (whole weeks)				
≤ 35	2		5%	
36	1		3%	
37	5		13%	
38	7		18%	
39	11		28%	
≥ 40	13		33%	
**Mean duration (SD) [CI]**	38 (4.5) [36.70–39.61]		100%	

*Note:* Demographics (ages, height, BMI ranges, weight changes, dietary habits, parity, and pregnancy duration for *n* = 39 pregnant and postpartum women. Results reported as mean (SD) [95% Confidence Interval] and number (*n*) and percent (%).

**TABLE 4 ∣:** Evaluation of adequacy for commonly used prenatal vitamins during 25–28 weeks gestation (A), 28–32 weeks gestation (B), and ≥ 3 months postpartum (C). “Amount provided” refers to nutrient amount the prenatal vitamin provides. Results reported as percentage of subject population with deficits left uncorrected after corresponding prenatal vitamin (PV A–H) was applied to nutritional content derived from subject-reported dietary food logs.

A	25–28 weeks gestation											
PV	A			B			C			D		
Nutrient	Amount provided	Percent still with deficit	95% confidence interval	Amount provided	Percent still with deficit	95% confidence interval	Amount provided	Percent still with deficit	95% confidence interval	Amount provided	Percent still with deficit	95% confidence interval
**Vitamin A (IU)**	2567	0%	(0%, 0%)	4000	0%	(0%, 0%)	3000	0%	(0%, 0%)	2167	0%	(0%, 0%)
**Vitamin C (mg)**	85	0%	(0%, 0%)	60	14%	(9.86%, 18.71%)	100	0%	(0%, 0%)	75	7%	(3.67%, 10.62%)
**Calcium (mg)**	150	79%	(77.25%, 81.08%)	200	71%	(68.59%, 73.08%)	140	79%	(77.25%, 81.08%)	75	92%	(90.45%,92.88%)
**Vitamin D (IU)**	1000	0%	(0%, 0%)	400	73%	(71.15%, 74.13%)	2000	0%	(0%, 0%)	1000	0%	(0%, 0%)
**Iron (mg)**	27	0%	(0%, 0%)	28	0%	(0%, 0%)	27	0%	(0%, 0%)	27	0%	(0%, 0%)
PV	E		F					G			H	
Nutrient	Amount provided	Percent still with deficit	95% confidence interval	Amount provided	Percent still with deficit	95% confidence interval	Amount provided	Percent still with deficit	95% confidence interval	Amount provided	Percent still with deficit	95% confidence interval
**Vitamin A (IU)**	1083	29%	(18.41%, 38.73%)	2167	0%	(0%, 0%)	0	100%	(100%, 100%)	2175	0%	(0%, 0%)
**Vitamin C (mg)**	20	50%	(45.18%, 54.82%)	120	0%	(0%, 0%)	0	100%	(100%, 100%)	65	14%	(9.86%, 18.71%)
**Calcium (mg)**	0	100%	(100%, 100%)	18	100%	(100%, 100%)	18	100%	(100%, 100%)	27	92%	(90.45%,92.88%)
**Vitamin D (IU)**	800	0%	(0%, 0%)	900	0%	(0%, 0%)	2000	0%	(0%, 0%)	600	3%	(2.01%, 4.05%)
**Iron (mg)**	0	100%	(100%, 100%)	0	100%	(100%, 100%)	0	100%	(100%, 100%)	50	0%	(0%, 0%)
B	28–32 weeks gestation											
PV	A			B			C			D		
Nutrient	Amount provided	Percent still with deficit	95% confidence interval	Amount provided	Percent still with deficit	95% confidence interval	Amount provided	Percent still with deficit	95% confidence interval	Amount provided	Percent still with deficit	95% confidence interval
**Vitamin A (IU)**	2567	0%	(0%, 0%)	4000	0%	(0%, 0%)	3000	0%	(0%, 0%)	2167	0%	(0%, 0%)
**Vitamin C (mg)**	85	0%	(0%, 0%)	60	33%	(29.36%, 37.31%)	100	0%	(0%, 0%)	75	11%	(7.93%, 14.29%)
**Calcium (mg)**	150	75%	(72.91%, 77.09%)	200	75%	(72.91%, 77.09%)	140	79%	(77.25%, 81.08%)	75	88%	(86.01%, 88.99)
**Vitamin D (IU)**	1000	0%	(0%, 0%)	400	87%	(85.88%, 87.87%)	2000	0%	(0%, 0%)	1000	0%	(0%, 0%)
**Iron (mg)**	27	0%	(0%, 0%)	28	0%	(0%, 0%)	27	0%	(0%, 0%)	27	0%	(0%, 0%)
PV	E		F					G			H	
Nutrient	Amount provided	Percent still with deficit	95% confidence interval	Amount provided	Percent still with deficit	95% confidence interval	Amount provided	Percent still with deficit	95% confidence interval	Amount provided	Percent still with deficit	95% confidence interval
**Vitamin A (IU)**	1083	11%	(4.75%, 17.47%)	2167	0%	(0%, 0%)	0	100%	(100%, 100%)	2175	0%	(0%, 0%)
**Vitamin C (mg)**	20	78%	(75.15%, 80.41%)	120	0%	(0%, 0%)	0	100%	(100%, 100%)	65	28%	(23.84%, 31.72%)
**Calcium (mg)**	0	100%	(100%, 100%)	18	100%	(100%, 100%)	18	100%	(100%, 100%)	27	100%	(100%, 100%)
**Vitamin D (IU)**	800	0%	(0%, 0%)	900	0%	(0%, 0%)	2000	0%	(0%, 0%)	600	3%	(1.80%, 3.46%)
**Iron (mg)**	0	100%	(100%, 100%)	0	100%	(100%, 100%)	0	100%	(100%, 100%)	50	0%	(0%, 0%)
C	≥ 3 months postpartum											
PV	A		B					C			D	
Nutrient	Amount provided	Percent still with deficit	95% confidence interval	Amount provided	Percent still with deficit	95% confidence interval	Amount provided	Percent still with deficit	95% confidence interval	Amount provided	Percent still with deficit	95% confidence interval
**Vitamin A (IU)**	2567	22%	(18.40%, 26.05%)	4000	0%	(0%, 0%)	3000	11%	(7.93%, 14.29%)	2167	44%	(40.58%, 48.31%)
**Vitamin C (mg)**	85	29%	(26.74%, 31.33%)	60	65%	(62.62%, 66.42%)	100	13%	10.96%, 14.84%)	75	52%	(49.46%, 53.77%)
**Calcium (mg)**	150	79%	(77.73%, 80.89%)	200	69%	(67.05%, 70.88%)	140	79%	(77.73%, 80.89%)	75	86%	(84.91%, 87.50%)
**Vitamin D (IU)**	1000	0%	(0%, 0%)	400	78%	(77.12%, 79.64%)	2000	0%	(0%, 0%)	1000	0%	(0%, 0%)
**Iron (mg)**	27	0%	(0%, 0%)	28	0%	(0%, 0%)	27	0%	(0%, 0%)	27	0%	(0%, 0%)
PV	E		F					G			H	
Nutrient	Amount provided	Percent still with deficit	95% confidence interval	Amount provided	Percent still with deficit	95% confidence interval	Amount provided	Percent still with deficit	95% confidence interval	Amount provided	Percent still with deficit	95% confidence interval
**Vitamin A (IU)**	1083	78%	(75.15%, 80.41%)	2167	44%	(40.58%, 48.31%)	0	100%	(100%, 100%)	2175	44%	(40.58%, 48.31%)
**Vitamin C (mg)**	20	94%	(92.72%, 94.38%)	120	0%	(0%, 0%)	0	100%	(100%, 100%)	65	58%	(56.03%, 60.10%)
**Calcium (mg)**	0	100%	(100%, 100%)	18	100%	(100%, 100%)	18	100%	(100%, 100%)	27	100%	(100%, 100%)
**Vitamin D (IU)**	800	0%	(0%, 0%)	900	0%	(0%, 0%)	2000	0%	(0%, 0%)	600	3%	(1.84%. 3.57%)
**Iron (mg)**	0	100%	(100%, 100%)	0	100%	(100%, 100%)	0	100%	(100%, 100%)	50	0%	(0%, 0%)

## Data Availability

The data that support the findings of this study are available from the corresponding author, Mary F. Hebert, upon reasonable request.
